# EBV-Related Diffuse Large B-Cell Lymphoma in a Patient with Angioimmunoblastic T-Cell Lymphoma

**DOI:** 10.4274/tjh.galenos.2018.2018.0023

**Published:** 2019-02-07

**Authors:** Cem Şimşek, Başak Bostankolu, Ece Özoğul, Arzu Sağlam Ayhan, Ayşegül Üner, Yahya Büyükaşık

**Affiliations:** 1 Hacettepe University Faculty of Medicine, Department of Internal Medicine, Ankara, Turkey; 2 Hacettepe University Faculty of Medicine, Department of Pathology, Ankara, Turkey; 3 Hacettepe University Faculty of Medicine, Division of Hematology, Ankara, Turkey

**Keywords:** Angioimmunoblastic T-cell lymphoma, Secondary lymphoma, EBV-related lymphoma, Cutaneous lymphoma

## To the Editor,

Angioimmunoblastic T-cell lymphoma (AITL) is a common subtype of peripheral T-cell lymphoma, accounting for approximately one-fifth of cases [1]. Epstein-Barr virus (EBV)-positive B cells are present in the tumor tissue in most cases [2]. Twenty-five cases of EBV-associated B-cell lymphomas in AITL patients have been reported in the literature [1,3,4,5,6,7,8,9,10,11,12,13,14,15]; herein, we report the 26^th^ case.

**Figure 1:**
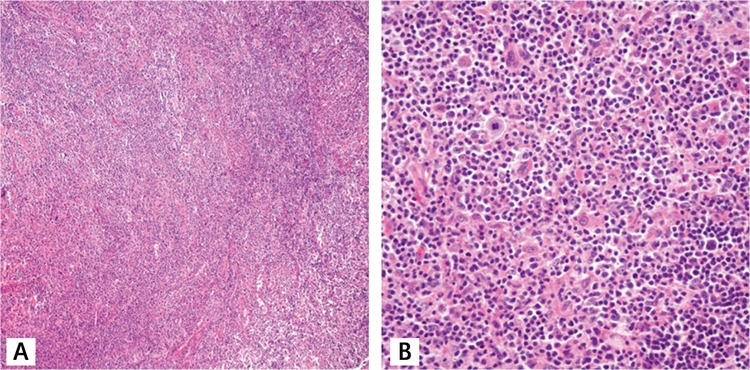
A, B) Cervical lymph node biopsy showing angoimmunoblastic T-cell lymphoma. Effacement of lymph node architecture, infiltrate of small to-medium sized lymphocytes, eosinophils, and occasional immunoblasts in a background of vascular proliferation consistent with angioimmunoblastic T-cell lymphoma.

**Figure 2:**
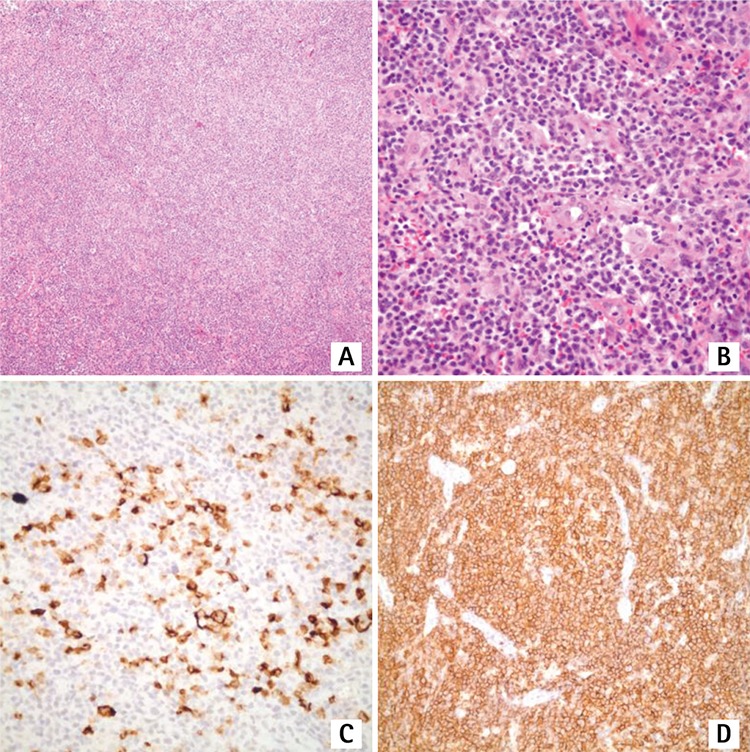
Biopsy from cutaneous lesion: A, B) infiltration of the dermis and subcutaneous tissue with large pleomorphic cells; C) with CD20 positivity; D) with LMP-1 positivity.

A 68-year-old female patient presented with B symptoms, multiple lymphadenopathies, and hepatosplenomegaly. Her laboratory studies were unremarkable except for normocytic anemia, eosinophilia, and increased lactate dehydrogenase. Her HIV serology was negative. Lymph node biopsy showed total effacement of the lymph node architecture with a polymorphic infiltrate composed of small to medium-sized lymphocytes, eosinophils, and occasional immunoblasts in a background of vascular proliferation (Figures 1A and 1B). Neoplastic cells were positive for CD3, CD4, PD1, and CD2. There was an extensive growth of follicular dendritic meshwork extending beyond the germinal centers. Large immunoblastic cells were scattered and positive for CD20. There were only occasional scattered LMP-1-positive blasts. However, T-cell receptor clonality analysis revealed a single prominent band with Tvγ-5J17 primers observed initially. In a subsequent biopsy, a sharp band with fr22 primers was observed. The morphologic and immunophenotypic features of the lymphoid proliferation were consistent with AITL. Six cycles of a CVP regimen (cyclophosphamide, vincristine, and prednisolone) were administered in 6 months. Doxorubicin or any other anthracycline was not administered due to associated cardiac morbidities. Post-treatment evaluation imaging showed radiological remission. However, nearly 1 month later, and 7 months after the initial diagnosis, she presented with multiple pruritic erythematous plaques on her arms and back. B symptoms had also returned. A biopsy from the largest lesion on her forearm showed infiltration of the dermis and subcutaneous tissue with large pleomorphic cells. Subsequent B-cell lymphoma cells that developed in the background of angioimmunoblastic lymphoma were positive for CD20, LMP-1, and EBER (Figure 2). The diagnosis was EBV-related diffuse large B-cell lymphoma (DLBCL) secondary to AITL. Control radiological examinations and/or bone marrow biopsies were not performed at that time. ICE (etoposide, iphosphamide, mesna, carboplatin) was started and 3 cycles were completed [16]. Rituximab (375 mg/m^2^ on day 1) was added to the protocol after the first cycle. A later control biopsy from skin lesions showed residual B-cell neoplasia. Control imaging also showed widespread lymphadenopathies in the neck, thorax, and abdomen. She was monitored closely without further cytotoxic treatment since she had poor performance status. The patient died of sepsis, with an overall survival of 14 months.

Secondary B-cell lymphoma may complicate AITL and has a poor prognosis. Only 3 of the previously reported 25 patients were described to have an overall survival longer than 12 months. Clinicians should be alerted by new-onset symptoms or lesions in a lymphoma patient, and suspicious lesions should be biopsied. The optimal treatment for either AITL or secondary DLBCL remains undefined.

Could the patient have had two different lymphomas (i.e. simultaneous or composite lymphomas) at the first presentation? It is impossible to exclude the possibility that she had additional EBV-related DLBCL in some of the multiple lymphadenopathies, with enlarged spleen and liver at presentation. Even so, the message to be taken from the association of AITL and EBV-related DLBCL (either simultaneous/composite or sequential) is the same: AITL is frequently EBV-positive and this positivity may result in EBV-positive DLBCL. Therefore, clinicians should be aware of this possibility.
